# Supplementary material for: Association between oral microbiome and seven types of cancers in East Asian population: a two-sample Mendelian randomization analysis
Source: Front Mol Biosci. 2023 Nov 21;10:1327893. doi: 10.3389/fmolb.2023.1327893 (PMC10702768; doi:10.3389/fmolb.2023.1327893)
Supplement: Supplementary file 1 [file DataSheet1.ZIP › Supplementary Materials/MR plots of saliva/saliva═╝/Prostate cancer/pheno.1504_to_prostate cancer_leave_one_out.pdf]

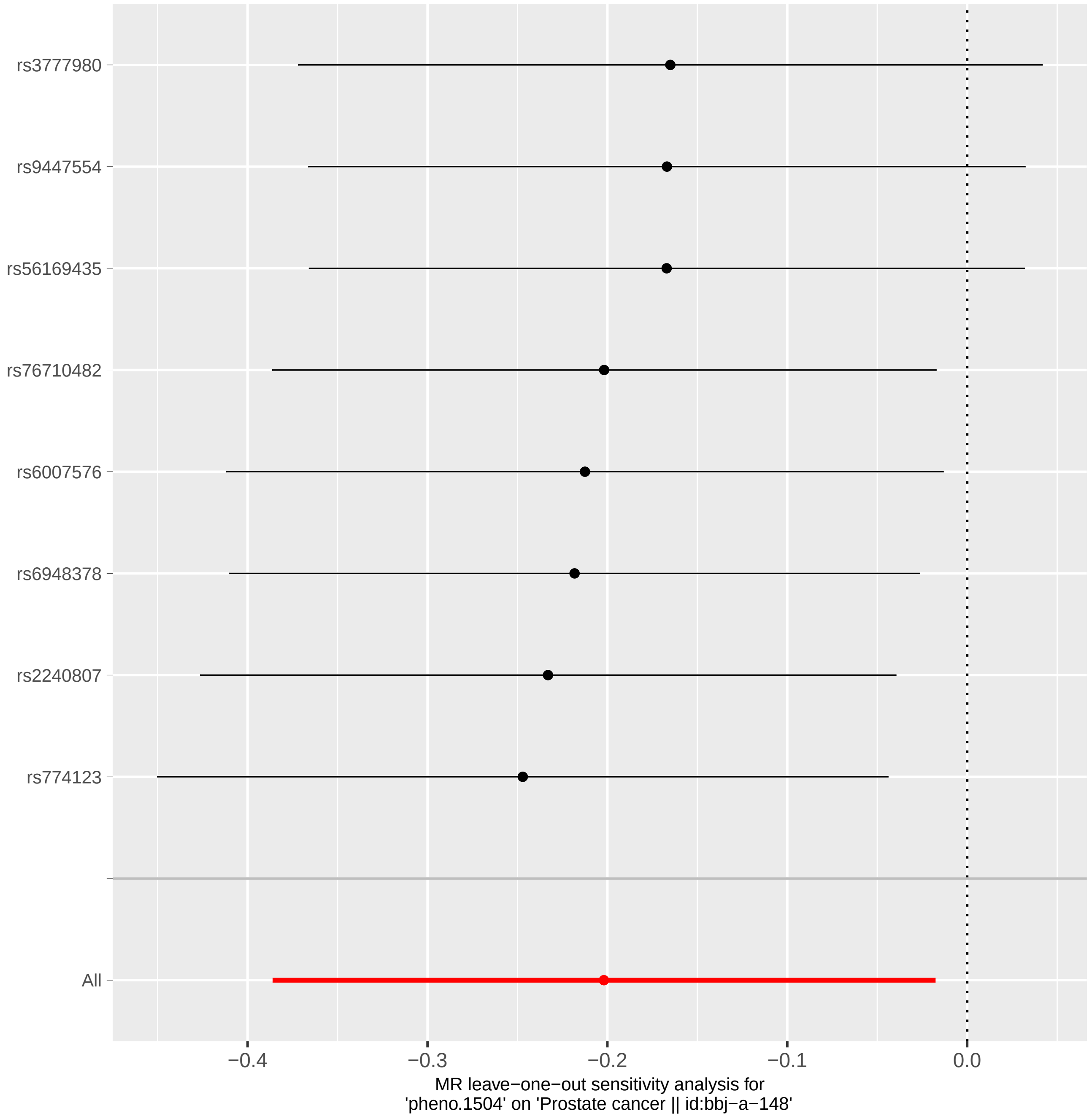
rs3777980
rs9447554
rs56169435
rs76710482
rs6007576
rs6948378
rs2240807
rs774123
All
−0.4
−0.3
−0.2
−0.1
0.0
MR leave−one−out sensitivity analysis for
'pheno.1504' on 'Prostate cancer || id:bbj−a−148'
